# Development and validation of an interpretable radiomic nomogram for severe radiation proctitis prediction in postoperative cervical cancer patients

**DOI:** 10.3389/fmicb.2022.1090770

**Published:** 2023-01-12

**Authors:** Chaoyi Wei, Xinli Xiang, Xiaobo Zhou, Siyan Ren, Qingyu Zhou, Wenjun Dong, Haizhen Lin, Saijun Wang, Yuyue Zhang, Hai Lin, Qingzu He, Yuer Lu, Xiaoming Jiang, Jianwei Shuai, Xiance Jin, Congying Xie

**Affiliations:** ^1^Wenzhou Key Laboratory of Biophysics, Wenzhou Institute, University of Chinese Academy of Sciences, Wenzhou, Zhejiang Province, China; ^2^The Second Affiliated Hospital of Wenzhou Medical University, Wenzhou, Zhejiang Province, China; ^3^Medical and Radiation Oncology, The Second Affiliated Hospital of Wenzhou Medical University, Wenzhou, Zhejiang Province, China; ^4^Radiotherapy Center, The First Affiliated Hospital of Wenzhou Medical University, Wenzhou, Zhejiang Province, China; ^5^School of Basic Medical Sciences, Wenzhou Medical University, Wenzhou, Zhejiang Province, China

**Keywords:** radiomics, nomogram, radiation proctitis, SHapley Additive exPlanation (SHAP), microbiota

## Abstract

**Background:**

Radiation proctitis is a common complication after radiotherapy for cervical cancer. Unlike simple radiation damage to other organs, radiation proctitis is a complex disease closely related to the microbiota. However, analysis of the gut microbiota is time-consuming and expensive. This study aims to mine rectal information using radiomics and incorporate it into a nomogram model for cheap and fast prediction of severe radiation proctitis prediction in postoperative cervical cancer patients.

**Methods:**

The severity of the patient’s radiation proctitis was graded according to the RTOG/EORTC criteria. The toxicity grade of radiation proctitis over or equal to grade 2 was set as the model’s target. A total of 178 patients with cervical cancer were divided into a training set (*n* = 124) and a validation set (*n* = 54). Multivariate logistic regression was used to build the radiomic and non-raidomic models.

**Results:**

The radiomics model [AUC=0.6855(0.5174-0.8535)] showed better performance and more net benefit in the validation set than the non-radiomic model [AUC=0.6641(0.4904-0.8378)]. In particular, we applied SHapley Additive exPlanation (SHAP) method for the first time to a radiomics-based logistic regression model to further interpret the radiomic features from case-based and feature-based perspectives. The integrated radiomic model enables the first accurate quantitative assessment of the probability of radiation proctitis in postoperative cervical cancer patients, addressing the limitations of the current qualitative assessment of the plan through dose-volume parameters only.

**Conclusion:**

We successfully developed and validated an integrated radiomic model containing rectal information. SHAP analysis of the model suggests that radiomic features have a supporting role in the quantitative assessment of the probability of radiation proctitis in postoperative cervical cancer patients.

## 1. Introduction

Cervical cancer is a malignant neoplasm at the junction of the squamous epithelial cells of the vaginal or transitional zone of the cervix and the endocervical canal columnar epithelial cells. Cervical cancer is the fourth most common cancer worldwide (Sung et al., 2021). Radiotherapy is one of the most effective methods for treating pelvic malignancies and has an irreplaceable role in treating cervical cancer at all stages. The main complication of radiotherapy for pelvic malignancies is radiation proctitis (Yeung et al., 2020). Fifty percent of patients with cervical cancer or endometrial cancer who received postoperative intensity-modulated radiotherapy developed acute rectal toxicity, and 5%–10% developed chronic rectal toxicity (Zelefsky et al., 2008; Yeung et al., 2020).

Unlike simple radiation damage to other organs, radiation proctitis is a complex disease closely related to the microbiota. A study using a rectal radiation mouse model showed that radiation affected both host and intestinal microbiota (Gerassy-Vainberg et al., 2018). Radiation therapy could induce local microbial ecological dysbiosis, and the dysbiosis microbiota could exert a direct pro-inflammatory effect on epithelial cells. In another study of 32 female patients with chronic radiation proctitis, differential patterns of dysbiosis were observed after analyzing the gut microbiota of patients with or without hematochezia (Liu et al., 2021). Gut microbiota could offer a set of biomarkers for radiation enteritis prediction, disease activity evaluation, and treatment selection (Wang et al., 2019).

However, the current prediction models of radiation proctitis were focused mainly on clinical and radiotherapy dose features. Several univariate and multivariate analyses showed that features, including tumor size, pathological characteristics, and radiological parameters, were significantly correlated with post-radiotherapy comorbidities in patients undergoing pelvic radiotherapy (Albert et al., 2008; Schmidt et al., 2022). A study by Fiorino et al. showed that rectal function was significantly correlated with treatment volume, PTV margins, radiation therapy dose, hemorrhoids presence, anticoagulant use during follow-up, and relative (%) and absolute (cm^3^) values of rectal V38Gy and V40Gy correlated with rectal bleeding (Fiorino et al., 2008). A study by Mahal et al. also noted that total radiation dose, dose per fraction, radiotherapy techniques, and treatment volume affected the rectum of patients (Mahal et al., 2014).

Another review also suggested features associated with radiation proctitis, including vascular diseases such as smoking, diabetes, hypertensive diabetes and atherosclerosis, collagen vascular disease, comorbid inflammatory bowel disease, and human immunodeficiency virus infection. Also, the review noted that specific underlying genetic changes could affect patients’ sensitivity to radiation. There was a correlation between genes and higher risks of gastrointestinal and genitourinary tract radiotoxicity after radiotherapy (Shadad et al., 2013).

The above studies have shown a strong correlation between patients’ oncologic features, pathologic features, and radiologic dose and the appearance of radiation proctitis in postoperative radiotherapy for pelvic cancer. However, the conclusions of these studies are inconsistent, and the accuracy of the prediction of radiation proctitis is unsatisfactory.

In recent years, computer-aided diagnosis, especially machine learning methods, has also been used for postoperative radiotherapy side effects and comorbidities prediction in oncology patients. Lee et al. applied machine learning methods such as random forest and bioinformatics to genome-wide data to predict and interpret advanced urogenital toxicity (Lee et al., 2018). They designed more robust predictive models and identified plausible biomarkers and biological processes associated with late urogenital toxicity. A study by Lewis & Kemp et al. showed that the integration of machine learning and genome-scale metabolic modeling identified multi-group biomarkers of radiation resistance (Lewis and Kemp, 2021).

However, it should be noted that the machine learning models above were developed using clinical features only. It ignored the large number of features embedded in computed tomography images (CT) that are imperceptible to the naked eye. Moreover, in the process of treatment plan determination, physicians are more focused on extracting focal area information and lack awareness of pelvic rectal information. Therefore, a comprehensive model is urgently needed to deepen the understanding of patient rectal image information to accurately assess radiotherapy treatment plans and reduce severe complications of radiation proctitis.

Deep learning, as well as dynamical modeling, is demonstrating powerful feature extraction and modeling capabilities in various medical fields (Li et al., 2021; Qian et al., 2021; Chen et al., 2022; Hu et al., 2022; Li Y. et al., 2022; Li X. et al., 2022). In data-driven disease research, a graph neuro network was used to predict the potential associations of disease-related metabolites (Sun et al., 2022). Deep learning can also be used to explore the identification of circRNA-disease associations (Wang et al., 2021) and predict the potential human lncRNA interactions (Zhang et al., 2021; Jingxuan et al., 2022; Wang et al., 2022). In drug metabolism research, deep learning can be used to predict the ability of a compound to permeate across the blood–brain barrier (Tang et al., 2022) and drug response (Kuenzi et al., 2020). At the same time, deep learning is also a popular tool for radiotherapy research. Zhong et al. developed a deep learning-based radiomic nomogram that could predict the prognosis of patients with different treatment regimens (Zhong et al., 2021). Qiang et al. established a prognosis prediction system based on deep learning for locoregionally advanced nasopharyngeal carcinoma (Qiang et al., 2021). Although deep learning has been widely applied in the analysis and prediction of various diseases, the poor interpretability of the deep learning model makes it difficult for clinicians to understand and trust these tools (Huff et al., 2021).

Radiomics can extract biomedical images containing information reflecting the underlying pathophysiology and reveal the relationships through quantitative image analysis (Le et al., 2021; Lam et al., 2022). In previous studies, radiomics has been used to predict postoperative radiotherapy-induced toxicity in prostate cancer patients. Mostafaei et al. showed that models based on CT radiomics, clinical features, and dose-volume parameters could predict radiation toxicity. The combination of imaging and clinical features could improve the performance of radiotoxicity prediction models (Mostafaei et al., 2020). However, no study has been performed to predict postoperative radiotherapy comorbidity in cervical cancer patients using radiomic features. Due to the limited resolution, the information on microbiota is almost impossible to extract directly by radiomics in theory, and no relevant studies have been reported. However, it is feasible that radiomics can indirectly reflect the effect of microbiota on the rectum.

Therefore, this study uses radiomics to extract the rectal information from medical images and improve the model performance and diagnostic accuracy through quantitative image analysis. Moreover, this study creatively introduces SHapley Additive exPlanation (SHAP) values to explore the interpretability of nomogram to improve the clinicians’ understanding of the model and its radiomic features, which facilitates later clinical promotion.

## 2. Materials and methods

### 2.1. Patients

The study protocol was reviewed and approved by the Ethics Committee in Clinical Research (ECCR) of the First Affiliated Hospital of Wenzhou Medical University. It was conducted following the Declaration of Helsinki. The Transparent Reporting of Individual Prognosis or Diagnosis Multivariate Predictive Models (TRIPOD) guidelines and the Strengthening Reports of Observational Studies in Epidemiology (STROBE) statement were applied. As this study was a retrospective cohort study, informed consent was waived, and all patient data were anonymized and desensitized.

Patients with cervical cancer from 1st January 2015 to 31st December 2020 in the First Affiliated Hospital of Wenzhou Medical University were collected for this study. These patients received a cervical cancer diagnosis, oncological surgery, and postoperative radiotherapy at the First Affiliated Hospital of Wenzhou Medical University.

**Figure 1:**
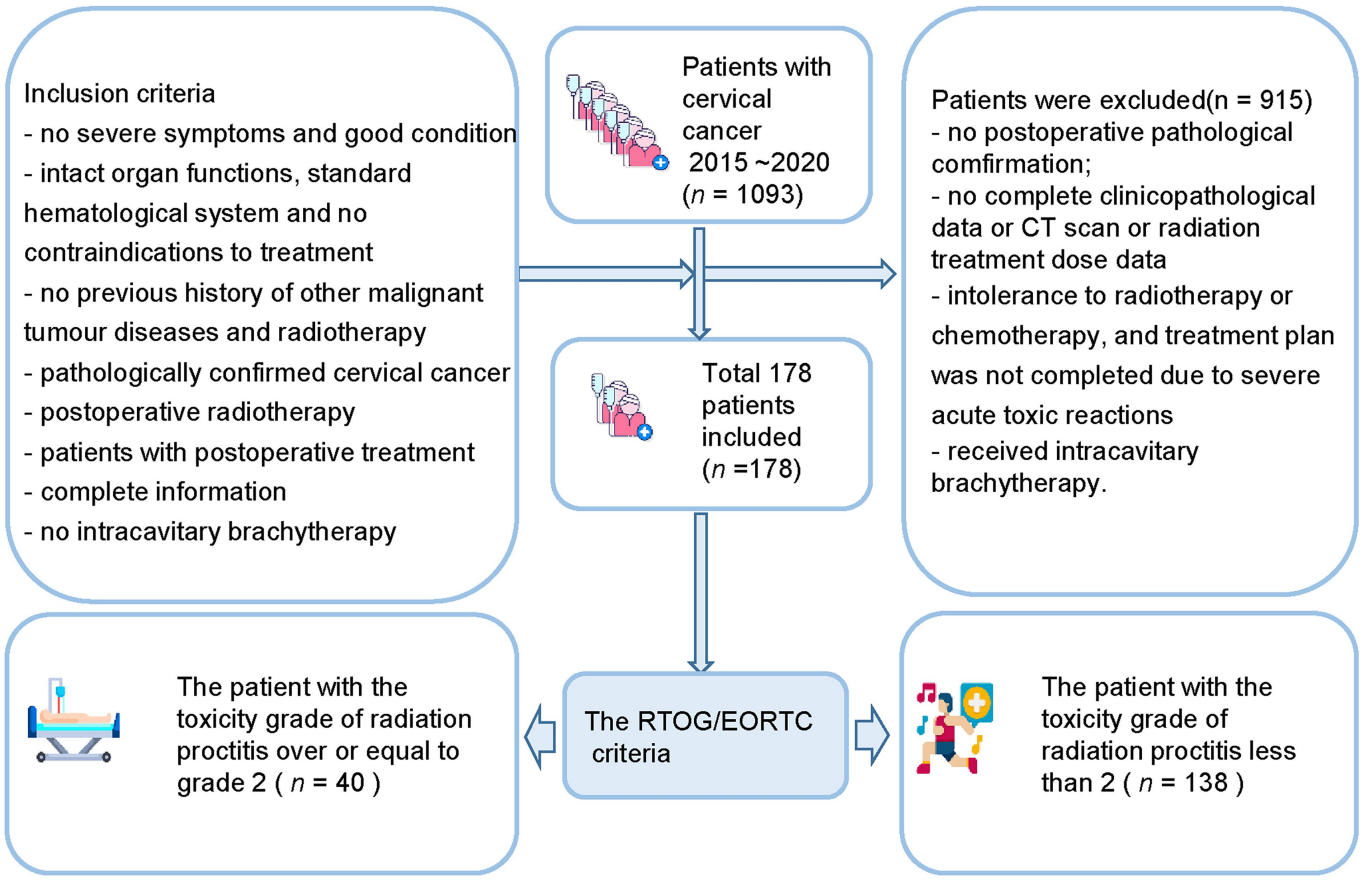
Flow diagram of the study enrolment patients.

The inclusion criteria (Figure 1) include (a) no severe symptoms at the time of diagnosis and good general physical condition; (b) patients with relatively intact organ functions, basically standard hematological system, and no contraindications to treatment; (c) no previous history of other malignant tumor diseases and radiotherapy; (d) postoperative pathological examination results confirming the diagnosis of cervical cancer; (e) postoperative radiotherapy; (f) patients with postoperative treatment; (g) the patient had complete pathology, imaging, and radiation therapy dose information; (h) no intracavitary brachytherapy was performed.

The exclusion criteria include (a) no definite postoperative pathological findings; (b) no complete clinicopathological data; (c) no CT scan was performed before postoperative radiotherapy; (d) patient’s pathology, imaging, and radiation treatment dose data are missing; (e) intolerance to radiotherapy or chemotherapy, and treatment plan was not completed due to severe acute toxic reactions during treatment; (f) Have received intracavitary brachytherapy.

The Mann–Whitney *U*-test and the Chi-square test were used to evaluate the performance of clinical and dose-volume features. Patients were randomly divided into a training set and a validation set.

### 2.2. Extraction of radiomic features

The entire rectal region on the patient’s CT image was defined as the Regions of Interest (ROI). Using ITK-SNAP software (Yushkevich et al., 2006), a pelvic radiologist with 10 years of experience at the First Affiliated Hospital of Wenzhou Medical University outlined this target region manually. Another radiologist with 20 years of experience reviewed it. Extraneous components other than the rectum, such as peripheral vessels, peripheral tissues, and peripheral organs, were not outlined by radiologists to minimize interfering information. The two radiologists did not know the patient’s information. If the two doctors had the same opinion, the ROI would be included in the imaging data set.

**Figure 2:**
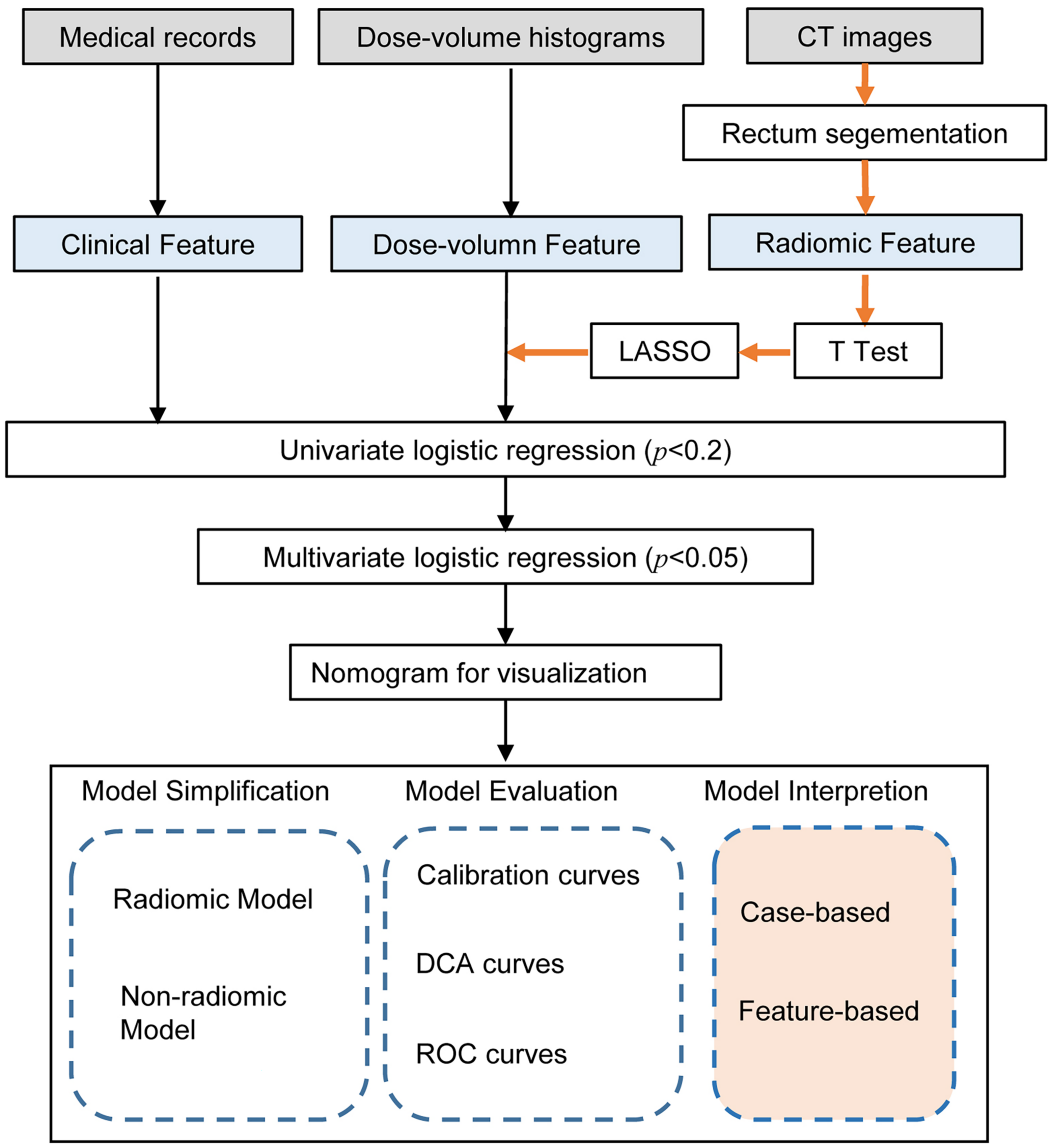
Workflow of the radiomic model development and model analysis process. The orange arrows in the flow chart represented the processing of the radiomic features. The radiomic features were generated by the PyRadiomics package after outlining the rectal region on the original image. After feature selection by *T*-test, LASSO and univariate logistic regression, multivariate logistic regression models were developed and visualized as nomograms. The model analysis consists of three parts: model simplification, model evaluation, and model interpretation. By comparing the performance change before and after model simplification, we could measure the importance of the radiomic features. In particular, we applied SHapley Additive exPlanation (SHAP) values for the first time to a radiomics-based logistic regression model to further interpret the radiomic features from case-based and feature-based perspectives.

Quantitative radiological features were automatically extracted using a feature extraction platform based on the Python package PyRadiomics (van Griethuysen et al., 2017). After segmentation and reconstruction of the patient CT, each patient extracted ROI was imported into Python in nrrd format. We extracted 1,409 radiomic features, including 8 feature classes used for further analysis and regression modeling. Radiomics features were dependent on the CT hardware, scanning parameters, and contrast agents. The process of generation and selection of radiomic features was illustrated in Figure 2.

### 2.3. Feature selection and model development

The variance equality of radiomics features was assessed by Levene’s test. Independent t-test or Wilcoxon’s test was used for feature selection. After standardizing the radiomic features using the z fraction transform, the high-dimensional imaging features extracted from the ROI were selected by the least absolute shrinkage and selection operator (LASSO) regularization algorithm. We performed univariate logistic regression on all features to screen out the key features significantly associated with the severity of radiation proctitis. The value of *p* was usually set at *p* < 0.2, but can also be set at *p* < 0.05 or *p* < 0.1. It requires the researcher to adjust the value of *p* according to the sample size. Due to the limited amount of data in this study, we set *p* < 0.2 as the threshold. Features with *p* > 0.2 in the univariate logistic regression were excluded, and features with *p* < 0.2 were included. Finally, the features left after multiple screenings were introduced into a stepwise logistic regression analysis to build a comprehensive model.

### 2.4. Model simplification and model evaluation

The critical features remained after multiple screenings were included in the multivariate logistic regression model generated by the stepwise forward and backward methods. Finally, to transform the complex regression equations into simple and visual graphs and make the prediction models’ results more readable, a visual nomogram was constructed based on these features that can be stably present in the unified model. All model evaluations were performed on the unseen validation set. In addition, calibration curves were used to evaluate the model performance of the nomogram.

To further validate the performance of the radiomic features, we built a simplified non-radiomic model by removing the radiomic features. To evaluate the performance of these two models, we assessed the discrimination using the receiver operating characteristic (ROC) analysis. The area under the receiver operating characteristic curve (AUC) was used to assess the predictive discrimination of these two models. In addition, in order to verify the validity of the model from another perspective, a k-Nearest Neighbor (KNN) model was built using the same data as the radiomic nomogram. The root-mean-square error (RMSE) and 10-fold cross-validation were used to select the optimal hyperparameter of the KNN model. We used decision curve analysis (DCA) to assess clinical validity by quantifying the net benefit at each threshold probability. All statistical analyses were performed using R (version 4.2.2), Python (version 3.9.12), and SPSS (version 24.0, IBM). The workflow of the model analysis process after modeling was shown in Figure 2.

### 2.5. Model interpretation

SHapley Additive exPlanation (SHAP) method is a game-theoretic-based model interpretation method. From a game theory perspective, SHAP treats each feature variable as a player. The predicted outcome obtained by the model is considered as the gain from the cooperation of many players to complete a project. It connects optimal credit allocation with local explanations using the classical Shapley values from game theory and their related extensions (Lundberg and Lee, 2017). We used scikit-learn (Pedregosa et al., 2011) to build the logistic regression model and used the SHAP package to calculate the SHAP values for the logistic regression model and further analyze the SHAP values with the SHAP plot module. The decision process of each patient could be presented by force plot. By overlaying the force plots and sorting the output values, we could see how all patients made their decisions. In addition to analyzing the model from the patient’s perspective, we can also use SHAP to understand the model from the feature’s perspective. SHAP provides bar plots and scatter plots of features to help us understand which feature was most important to the model.

## 3. Results

### 3.1. Baseline information of patients

This study included 1,093 patients with cervical cancer who needed to initiate radiotherapy at the First Affiliated Hospital of Wenzhou Medical University between 1st January 2015 and 31st December 2020. After screening and exclusion, a total of 178 patients were finally included in our study. The study included 40 patients (22.5%) with a toxicity grade greater than or equal to grade 2 after radiation therapy and 138 patients (77.5%) less than grade 2 after radiation therapy. The patients were divided into a training set (*n* = 124) and a validation set (*n* = 54). Table 1 shows the baseline information of the patients.

**Table 1:** Baseline information of all patients.

Variables		Primary queue (*n* = 178)		
		<grade 2 (*n* = 138)	≥grade 2 (*n* = 40)	*p*-value
Age (years)		53.5 (46–61)	52(47.75–60.75)	0.957
Therapy	3D-CRT	53 (63.9%)	30 (36.1%)	<0.001
	VMAT	85 (89.5%)	10 (10.5%)	
Vascular invasion		48 (69.6%)	21 (30.4%)	0.043
FIGO Staging		2(1–2)	1(1–2.75)	0.800
Total rectal volume		65.937 (51.421–94.235)	69.422 (51.921–89.546)	0.875
Minimum rectal dose		1320.85 (417.6–2205.55)	583.35 (407.775–1817.025)	0.189
Maximum rectal dose		4907.35 (4145.175–5293.575)	4187.7 (4136.525–4819.25)	0.038
Average rectal dose		3958.55 (3738.35–4144.6)	3928.8 (3831.325–3996.2)	0.289
V5Gy(cm^3^)		63.444 (47.286–89.492)	69.422 (51.921–89.546)	0.427
V5Gy(%)		100 (98.148–100)	100(99.555–100)	0.987
V10Gy(cm^3^)		62.984 (47.127–90.466)	69.422 (53.625–92.057)	0.260
V10Gy(%)		99.95 (95.415–100)	99.245 (98.365–100)	0.976
V15Gy(cm^3^)		62.511 (46.688–90.466)	69.422 (53.625–91.919)	0.283
V15Gy(%)		99.18 (93.693–100)	98.52 (97.268–100)	0.837
V20Gy(cm^3^)		62.511 (46.234–89.878)	69.422 (53.625–90.757)	0.274
V20Gy(%)		97.755 (92.235–100)	97.855 (95.11–99.743)	0.705
V25Gy(cm^3^)		61.865 (45.854–87.485)	68.366 (53.122–88.878)	0.240
V25Gy(%)		95.475 (89.273–99.065)	96.645 (92.355–98.093)	0.304
V30Gy(cm^3^)		59.571 (42.243–80.618)	67.518 (52.789–87.533)	0.181
V30Gy(%)		90.145 (84.488–95.985)	95.06 (87.873–97.298)	0.028
V35Gy(cm^3^)		56.05 (39.968–76.027)	61.987 (48.092–86.923)	0.203
V35Gy(%)		83.955 (75.073–91.188)	92.34 (84.098–95.993)	0.001
V40Gy(cm^3^)		42.149 (27.754–60.107)	50.823 (33.909–65.466)	0.108
V40Gy(%)		59.66 (50.4–70.375)	65.595 (58.38–76.108)	0.035
V45Gy(cm^3^)		16.175 (0–30.87)	0(0–15.492)	0.003
V45Gy(%)		26.465 (0–38.913)	0(0–16.76)	0.001

### 3.2. Radiomic features selection and multivariate analysis

We extracted a total of 1,409 radiomic features from the patients’ CTs and selected them using the LASSO algorithm. Multivariate logistic regression analysis was performed on all features selected by LASSO and univariate logistic regression. The results of the multivariate logistic regression are shown in Table 2. Radiotherapy techniques [OR = 0.000 (0.000–0.086), *p* = 0.005], Maximum rectal dose [OR = 1.006 (1.001–1.011), *p* = 0.020], Contrast [OR = 0.000 (0.000–0.002), *p* = 0.046] were independent risk factors for severe radiation proctitis.

**Table 2:** Result of multivariate logistic regression.

Features	B	*P*	OR (95% CI)
Therapy	−8.225	0.005	0.000(0.000–0.086)
Maximum rectal dose	0.006	0.020	1.006(1.001–1.011)
Contrast	−349.316	0.046	0.000(0.000–0.002)
Constant	−23.030	0.026	0.000

### 3.3. Establishment of nomogram and model evaluation

**Figure 3:**
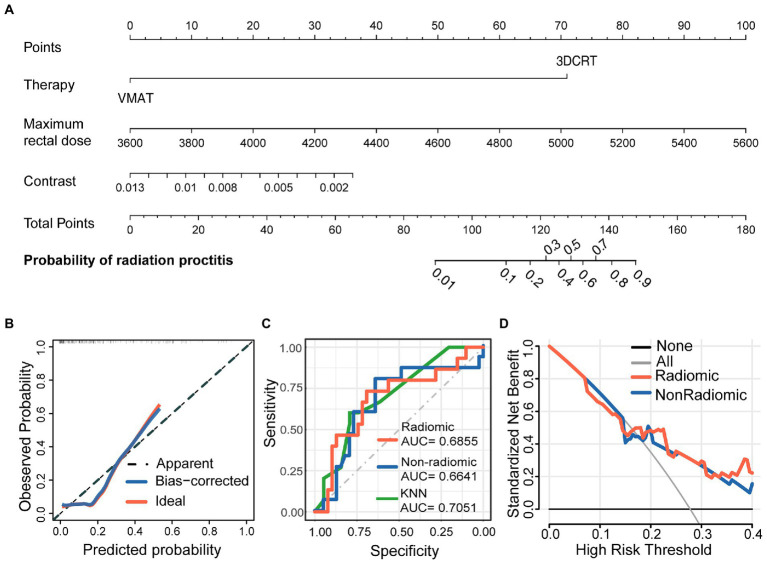
Nomogram for severe radiation proctitis prediction in postoperative cervical cancer patients, calibration of the nomogram, and decision curves in the overall patients. The combined nomogram **(A)** incorporated clinical, dose-volume, and radiomic features. By accumulating the points for each feature, we could quickly calculate the probability of radiation proctitis. All model evaluations were performed on the validation set. The Calibration curves of the combined radiomics nomogram **(B)** illustrated the relationship between the observed outcome frequencies and the predicted probabilities. The ROC curves **(C)** demonstrated the accuracy of the radiomic and non-radiomic models and KNN radiomic model. The DCA curves **(D)** demonstrated the net benefit of the radiomic and non-radiomic models.

In order to develop a clinically applicable method to predict the occurrence of radiation proctitis, we constructed a radiomics nomogram. The results of the nomogram were shown in Figure 3A. All model evaluations were performed on the unseen validation set. The calibration curve of the combined radiomics nomogram was shown in Figure 3B. To further validate the performance of the radiomic features, we built a simplified non-radiomic model based only on the clinical feature and dose-volume feature by removing the radiomic feature and comparing its performance with the full radiomic model. The ROC curves for the two nomogram models (Figure 3C) showed that the prediction effect of the radiomic model [AUC = 0.6855 (0.5174–0.8535)] performed better than the non-radiomic model [AUC = 0.6641 (0.4904–0.8378)]. The AUC of radiomic nomogram [AUC = 0.6855 (0.5174–0.8535)] was close to that of the KNN model [AUC = 0.7051 (0.5602–0.85)]. It illustrated the validity of the model from another perspective. The decision curve analysis (DCA; Figure 3D) was used to assess the utility of both prediction models by calculating the net benefit at various probability thresholds. According to the decision curves, the radiomic model showed more benefit in predicting the risk of radiation proctitis than the non-radiomic model. It suggested that radiomic features were supporting features for severe radiation proctitis prediction.

### 3.4. Model interpretation

#### 3.4.1. Case-based model interpretation

**Figure 4:**
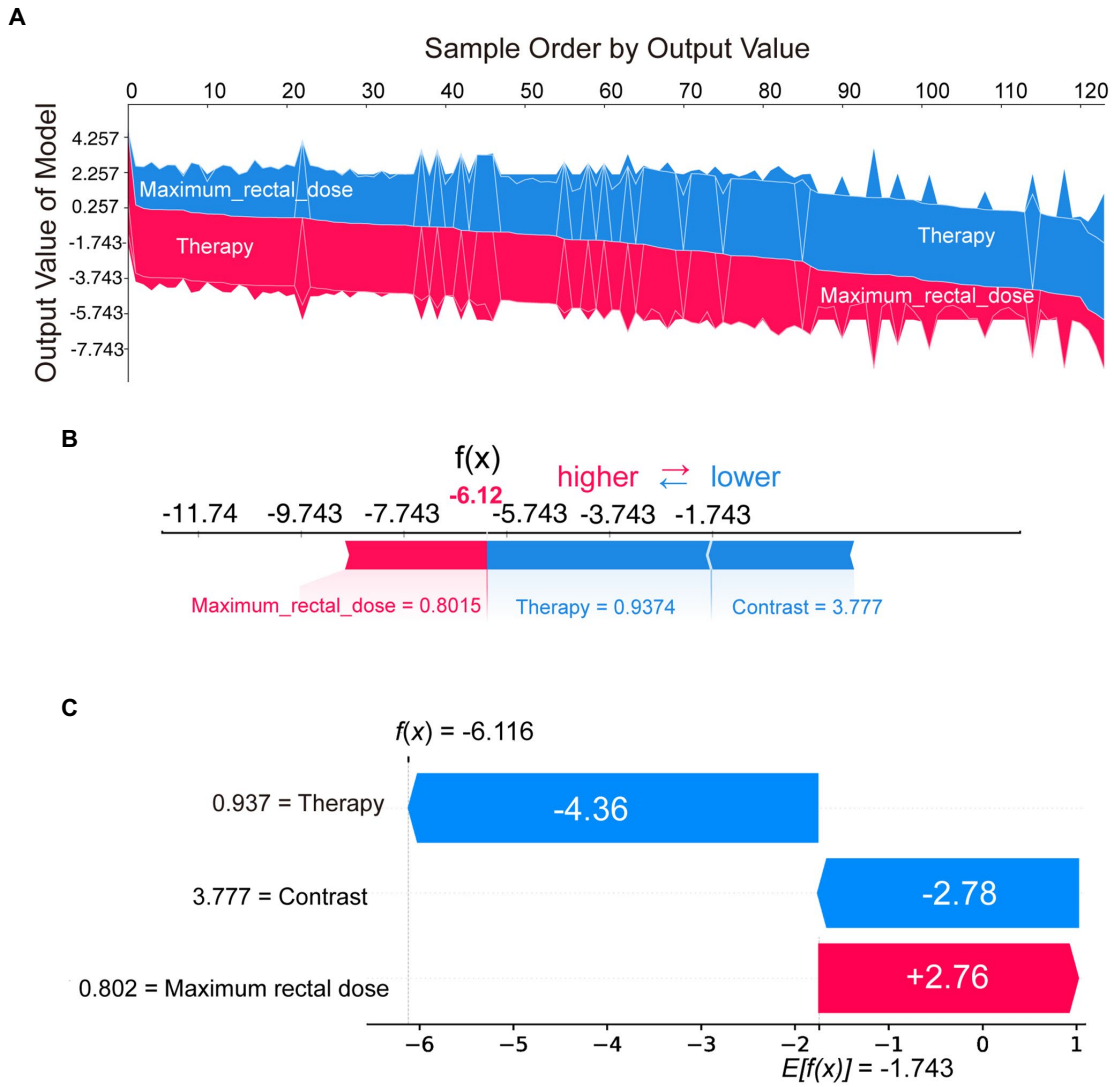
SHAP plots demonstrated SHAP values from a case-based perspective. Sampled by model output, the overall SHAP plot **(A)** showed the decision process of all patients. The force plot **(B)** and the waterfall plot **(C)** demonstrated the proportion and absolute SHAP value of various features in the decision-making process for a single patient.

To further understand how decision-making occurred for individual and entire patient populations, we used SHAP to analyze from a case-based perspective. Figure 4A represented the decision process for SHAP values across all patients, with the vertical axis representing the magnitude of the SHAP values. As the graph was ordered by model output, we could clearly see the boundary line between red and blue. Features pushing the prediction higher were shown in red, and those pushing the prediction lower were in blue.

In addition to the model interpretation for all cases, we could also provide a clearer picture of the decision-making situation for individual patients through the waterfall or force plot. For example, by selecting the patient on the far right of Figure 4A, the decision-making process could be visualized in Figure 4B or Figure 4C. Although the presentation was different, the information in Figures 4B,C was consistent. These two plots indicated the proportion and absolute SHAP value of various features in the decision-making process for that patient. SHAP could provide a quantitative and visual representation of the decision mechanisms of the radiomics model for any patient.

#### 3.4.2. Feature-based model interpretation

**Figure 5:**
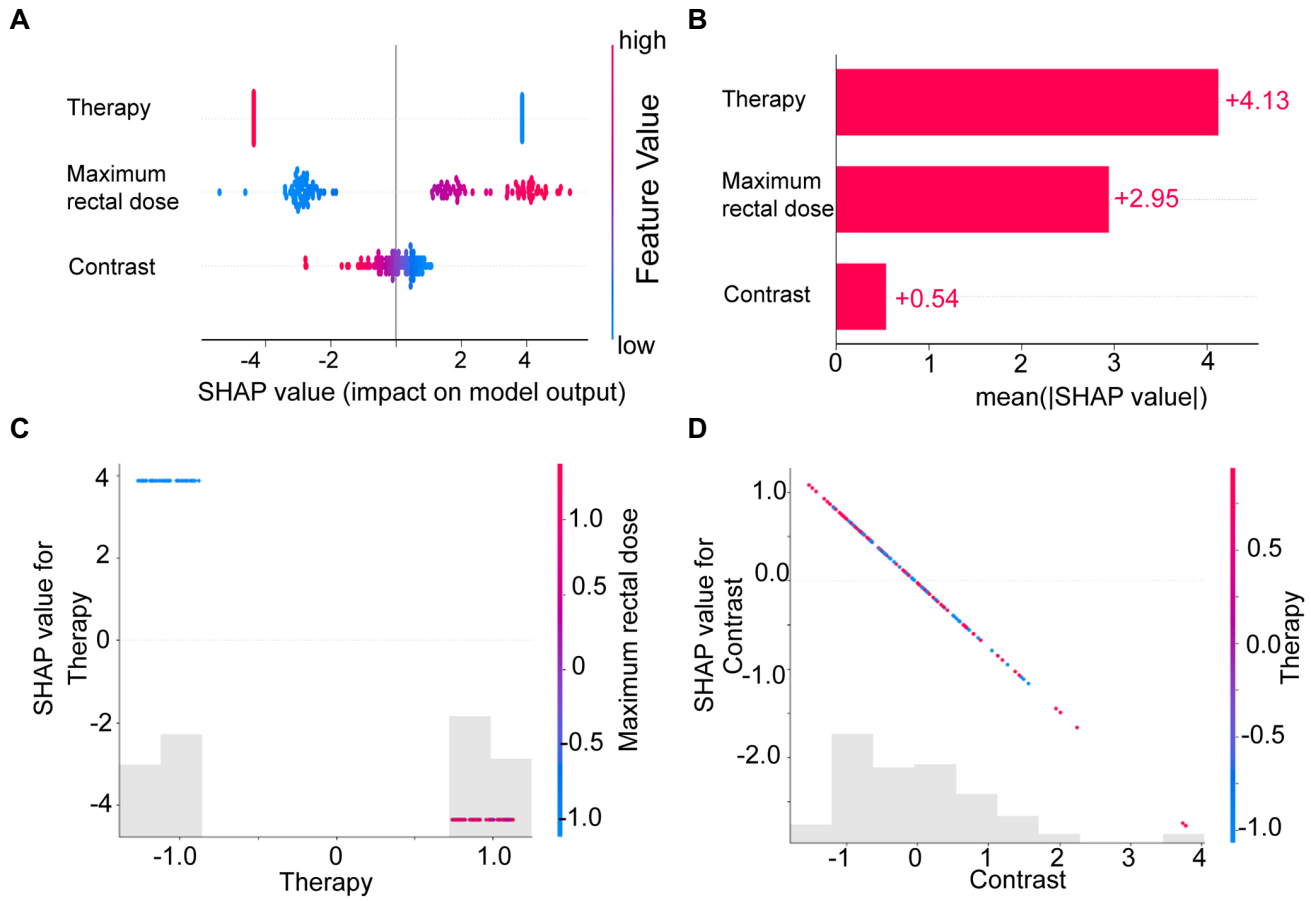
The SHAP plots illustrated the feature-based model interpretation process. **(A)** The beeswarm plot used SHAP values to show the distribution of each feature’s impacts. **(B)** The standard bar plot demonstrated the mean absolute value of the SHAP values for each feature. These two plots **(C,D)** showed the SHAP values in different features. Gray bar plots showed the SHAP values for each feature. Scatter plots showed the SHAP values of the other features most relevant to that feature. Vertical dispersion represented interaction effects between the horizontal and vertical features.

We calculated and visualized the SHAP values for each feature in the radiomics model. The beeswarm plot (Figure 5A) demonstrated an overview of the feature contribution of all patients. In the beeswarm plot, features were sorted by the sum of SHAP value magnitudes over all samples, and SHAP values were used to show the distribution of each feature’s impacts. The bar plot shown in Figure 5B demonstrated the mean absolute value of the SHAP values for each feature. The plot showed that radiotherapy techniques and the maximum rectal dose have a high mean value. Since SHAP values represented a feature’s responsibility for a change in the model output, Figures 5A,B indicated that the radiotherapy technique and the maximum rectal dose were essential.

To understand how each feature affected the model’s output, we plotted gray bar plots to show the SHAP values for each feature and scatter plots to show the SHAP values of the other features most relevant to that feature (Figures 5C,D).

## 4. Discussion

With the advancement of radiotherapy techniques, the postoperative survival rates of cancers such as cervical cancer have increased dramatically (Citrin, 2017). However, complications and side effects caused by postoperative radiotherapy or chemotherapy are difficult to avoid. Radiation proctitis is one of the most common complications of postoperative radiotherapy in patients with pelvic tumors (Rustagi et al., 2015; Qian et al., 2021), with mild diarrhea or mild rectal exudate in mild cases and even intestinal necrosis or bleeding in severe cases, endangering patients’ lives (Citrin, 2017). In clinical practice, doctors currently rely on the dose-volume features of radiotherapy plans to assess the risk of radiation proctitis. However, there is a lot of valuable information in pathology and clinical imaging that is not considered by clinicians. Moreover, the sensitivity of the rectum to radiotherapy radiation also varies significantly between individuals.

To further refine the assessment of radiation proctitis, we selected radiomic features associated with radiation proctitis by univariate regression and the LASSO algorithm. Radiotherapy techniques (OR = 0.000 (0.000–0.086), *p* = 0.005), Maximum rectal dose (OR = 1.006 (1.001–1.011), *p* = 0.020), Contrast (OR = 0.000 (0.000–0.002), *p* = 0.046) were independent risk factors for radiation proctitis. Finally, we developed an integrated prediction model based on clinical and radiomic features [AUC = 0.6855 (0.5174–0.8535)]. Current studies of radiation proctitis had mainly focused on local radiotherapy dose limits rather than comprehensive predictive models (Snyder et al., 2001; Huang et al., 2004). There was only one study using radiomics to build a predictive model for radiation proctitis (Mostafaei et al., 2020). In gastrointestinal toxicities modeling, the AUC of radiomic model of their study was 0.71, which was relatively higher compared with our study. However, the study was conducted based on data from only 64 patients and was only suitable for patients with prostate cancer.

The radiomic features of the model potentially incorporated the effect of microbiota on rectal radiosensitivity. The model without radiomic features showed lower validity, while the model containing both radiomic features and clinical features showed better performance on the ROC curve. The change of net benefit in Figure 3D suggested that radiomic features had played a supporting role in predictive models. And as a measure of the local intensity variation, a larger contrast correlated with a greater disparity in intensity values among neighboring voxels. In our study, the contrast suggested that a lower tissue density compared to the surrounding tissue was associated with higher radiosensitivity.

In most cases, PyRadiomics followed the image biomarker standardization initiative (IBSI)'s definition of features. PyRadiomics development was also involved in the standardization effort by the IBSI team. Still, there were some differences between PyRadiomics and feature extraction as defined in the IBSI documents. Most notably were the differences in gray value discretization (just for the fixed bin size type) and resampling. In summary, the definitions of PyRadiomics and IBSI were slightly different, but did not represent one over the other. Moreover, IBSI was only an initiative, not a standard. For these reasons, IBSI would not significantly impact the reproducibility and validity of this study.

While SHAP was often used to explain features in machine learning algorithms and neural network models (Bang et al., 2021; Park et al., 2022; Shaji et al., 2022; Shi et al., 2022), SHAP analysis of logistic regression models had not yet been mentioned. Although logistic regression algorithms were simpler and more explicit than other machine learning algorithms and neural networks, logistic regression models were more challenging to understand than they may seem. Users could not directly measure the importance of features between continuous and categorical variables through odds ratio (OR) or coefficients (Table 2). In particular, for radiomic models, the significant variation in the magnitude of radiomic features made it more challenging to understand the actual decision-making process of the model through the coefficients and OR values of logistic regression. We wanted to help users better understand each feature’s role in the model. In subsequent clinical treatment, model users can further quantify the contribution of radiomic features in each model output.

To address this issue, we introduced SHAP for the first time to a radiomics-based logistic regression model, which further revealed the model’s decision-making mechanism (Figure 4A). The total contribution of SHAP for each feature included in the model was analyzed (Figure 5B). Radiotherapy techniques and the maximum rectal dose occupied vital positions in the model contribution. Notably, the SHAP value of the radiomic feature was the lowest. It suggested that the radiomic feature was weaker than the clinical feature and dose-volume feature. The SHAP could also analyze correlations between variables (Figures 5C,D). Correlations in SHAP values were observed between the three features. It may suggest an inter-collaborative relationship between variables in the model. However, this can only indicate a correlation between SHAP values, not between the values of the variables. In the subplot of therapy (Figure 5C), we can find that the most relevant variable was the maximum rectal dose. There was a harmful effect of maximum rectal dose in the decision-making process of these VMAT therapy samples. However, no fixed pattern was observed in the subplot of contrast (Figure 5D).

SHAP had a unique role in radiomics-based logistic models as a game-theoretic approach. SHAP helped us understand radiomic features that vary significantly in magnitude. Furthermore, SHAP provided a quantitative and visual representation of the decision mechanisms within the model for each patient.

We recommend that clinicians can reduce the value of the maximum rectal dose by modifying the plan when the model suggests that the current radiotherapy plan has a high probability of radiation proctitis. Clinicians can rely on interpretable models to precisely control the risk of the final plan to an acceptable level. Patients with cervical cancer can reduce unnecessary radiation doses and the incidence of radiation proctitis with the help of the comprehensive model.

## 5. Conclusion

We successfully developed and validated an integrated radiomic model containing rectal information in this study. The integrated radiomic model enables the accurate quantitative assessment of the probability of radiation proctitis in postoperative cervical cancer patients, addressing the limitations of the current qualitative assessment based on dose-volume parameters only. Based on the model output and SHAP values analysis, we suggest that clinicians can adjust the radiation dose to minimize the occurrence of severe radiation proctitis while not compromising the effectiveness of radiation therapy.

## Data availability statement

The raw data supporting the conclusions of this article will be made available by the authors, without undue reservation.

## Ethics statement

The studies involving human participants were reviewed and approved by the Ethics Committee in Clinical Research (ECCR) of the First Affiliated Hospital of Wenzhou Medical University. Written informed consent for participation was not required for this study in accordance with the national legislation and the institutional requirements.

## Author contributions

CW, XX, and CX conceived the project, developed the prediction method, designed and implemented the experiments, analyzed the result, and wrote the manuscript. XZ, SR, and XJin implemented the experiments and analyzed the result. QZ, WD, HaizL, SW, and YZ analyzed the result. HaiL, QH, YL, XJia, and JS contributed to the interpretation of the results. All authors contributed to the article and approved the submitted version.

## Funding

This work was supported by Zhejiang Engineering Research Center for Innovation and Application of Intelligent Radiotherapy Technology in the Second Affiliated Hospital of Wenzhou Medical University, Wenzhou key Laboratory of radiotherapy and Translational Research of Cancer (2021100848), and Wenzhou Science and Technology Bureau Y2020733. This work was also supported by the Ministry of Science and Technology of the People’s Republic of China under grant 2021ZD0201900, the National Natural Science Foundation of China under grant numbers 12090052 and 11874310, and Major Projects in Fujian Province under grant 2020Y4001.

## Conflict of interest

The authors declare that the research was conducted in the absence of any commercial or financial relationships that could be construed as a potential conflict of interest.

## Publisher’s note

All claims expressed in this article are solely those of the authors and do not necessarily represent those of their affiliated organizations, or those of the publisher, the editors and the reviewers. Any product that may be evaluated in this article, or claim that may be made by its manufacturer, is not guaranteed or endorsed by the publisher.

## Abbreviations

SHAP: SHapley Additive exPlanation
CT: computed tomography
ROI: Regions of Interest
LASSO: the least absolute shrinkage and selection operator regularization
DCA: decision curve analysis
AUC: area under curve
OR: odds ratio
ROC: receiver operating characteristic analysis
ECCR: Ethics Committee in Clinical Research
RTOG: Radiation Therapy Oncology Group
EORTC: European Organization for Research and Treatment of Cancer
